# *KLF4*^*K409Q*^–mutated meningiomas show enhanced hypoxia signaling and respond to mTORC1 inhibitor treatment

**DOI:** 10.1186/s40478-020-00912-x

**Published:** 2020-04-03

**Authors:** Niklas von Spreckelsen, Natalie Waldt, Rebecca Poetschke, Christoph Kesseler, Hildegard Dohmen, Hui-Ke Jiao, Attila Nemeth, Stefan Schob, Cordula Scherlach, Ibrahim Erol Sandalcioglu, Martina Deckert, Frank Angenstein, Boris Krischek, Pantelis Stavrinou, Marco Timmer, Marc Remke, Elmar Kirches, Roland Goldbrunner, E. Antonio Chiocca, Stefan Huettelmaier, Till Acker, Christian Mawrin

**Affiliations:** 1 Department of Neuropathology, Otto-von-Guericke University, Magdeburg, Germany; 2 Department of Neurosurgery, Brigham and Women’s Hospital, Harvard Medical School, Boston, USA; 3 Department of Neurosurgery, Center for Neurosurgery, Faculty of Medicine and University Hospital, University of Cologne, Cologne, Germany; 4 Institute of Molecular Medicine, Martin Luther University, Halle/Saale, Germany; 5 Department of Neuropathology, University Giessen, Giessen, Germany; 6 Department of Neuroradiology, University Hospital Leipzig, Leipzig, Germany; 7 Department of Neurosurgery, Otto-von-Guericke University, Magdeburg, Germany; 8 Department of Neuropathology, University Hospital Cologne, Cologne, Germany; 9 Laboratory for Non-invasive Imaging, DZNE, Magdeburg, Germany; 10 Pediatric Neuro-Oncology, Duesseldorf, Germany

**Keywords:** Meningioma, Mutation, K409Q, Hypoxia, HIF, Edema, KLF4

## Abstract

Meningioma represents the most common primary brain tumor in adults. Recently several non-*NF2* mutations in meningioma have been identified and correlated with certain pathological subtypes, locations and clinical observations. Alterations of cellular pathways due to these mutations, however, have largely remained elusive. Here we report that the Krueppel like factor 4 (*KLF4)-K409Q* mutation in skull base meningiomas triggers a distinct tumor phenotype. Transcriptomic analysis of 17 meningioma samples revealed that *KLF4*^*K409Q*^ mutated tumors harbor an upregulation of hypoxia dependent pathways. Detailed in vitro investigation further showed that the *KLF4*^*K409Q*^ mutation induces HIF-1α through the reduction of prolyl hydroxylase activity and causes an upregulation of downstream HIF-1α targets. Finally, we demonstrate that *KLF4*^*K409Q*^ mutated tumors are susceptible to mTOR inhibition by Temsirolimus. Taken together, our data link the KLF4^K409Q^ mediated upregulation of HIF pathways to the clinical and biological characteristics of these skull base meningiomas possibly opening new therapeutic avenues for this distinct meningioma subtype.

## Introduction

Meningioma, a tumor arising from arachnoid cap cells, represents the most common primary brain tumor in adults with an incidence of 8.14/100,000 [23]. The majority of meningiomas belongs to the benign WHO I° group, whereas up to 20% correspond to grade II or grade III meningiomas with increased histological malignancy [27]. Along with direct compression of brain tissue and cranial nerves a subset of meningiomas causes peritumoral brain edema (PTBE) which in turn can lead to neural deficits, epileptic seizures and life-threatening periprocedural complications [28]. To date, there are no pharmaceutical therapeutic options for meningiomas, and surgical removal remains the treatment of choice [11]. Meningiomas located at the skull base are particularly difficult to treat [7, 13]. Their proximity to crucial neural and vascular structures makes surgical removal challenging, underlining the need for alternative treatment options.

Bi-allelic *NF*2 inactivation is the most common and most studied genetic alteration found in meningiomas. In recent efforts however, several distinct non-*NF2* mutations linked to skull base meningiomas have been described [5, 43]. Of these, *SMO* mutations are associated with the anterior skull base, have been shown to have an impact on the onset of tumor recurrence [3, 42] and both *AKT1*^*E17K*^ and *SMO* are currently investigated as candidates for targeted therapy (ClinicalTrials.gov: NCT02523014). Investigations regarding *KLF4* are less advanced. While the combination of *KLF4* and *TRAF7* mutations defines the secretory subtype [5] and *KLF4*^*K409Q*^ tumors have been shown to be associated with larger peritumoral edema [43], the biological function and molecular mechanisms associated with the *KLF4*^*K409Q*^ mutation have not been elucidated.

The Krüppel-like factor 4 (KLF4) is a transcription factor involved in a variety of cellular signaling pathways. Its expression is induced by a variety of factors including inflammation, DNA damage and oxidative stress and its posttranslational regulation is largely dependent on Von Hippel-Lindau tumor suppressor (pVHL) induced degradation [8, 9]. KLF4 has been shown to regulate crucial pathways in cell differentiation, proliferation, inflammation and apoptosis. It is a critical factor in generating induced pluripotent stem cells and promoting angiogenesis via activation of VEGF [35, 38]. In the onco-genetic context, KLF4 acts as a tumor suppressor in colon cancer, but plays a potent oncogenic role in mammary carcinoma and melanoma. Its highly context-dependent function in tumor biology renders it a challenging, but in many tumor-entities promising therapeutic target [31, 33, 34, 36, 39]. Indeed, some KLF4 inhibitors, for instance Statins, have been already identified and recently characterized as promising inhibitory agents in osteosarcoma [6, 19, 44].

Previous data have shown that about 10–14% of meningiomas harbor the mutation *KLF4*-p.K409Q (*KLF4*^K409Q^) [5, 29]. This missense mutation is highly specific for meningiomas and has only been reported in four non-meningeal tumors (two pancreatic and two breast derived cancers) [12].

Here we demonstrate that *KLF4*^*K409Q*^ mutation in meningioma leads to an upregulated HIF-1α pathway, leaves cells susceptible to hypoxia and that this effect can be blocked by mTOR inhibition with Temsirolimus.

## Materials and methods

### Clinical data and human specimens

Clinical data and Tumor material from 96 meningioma patients were collected and analyzed (study was approved by the local ethical committee (Application No. 03–170)).

### Statistics

Statistical analysis was performed using SPSS, release 22 and GraphPad Prism 7. ANOVA, t-test, Chi-square and Fisher’s exact where used for gaussian distributed data, Mann-Whitney-U and Kruskal-Wallis when data did not meet the normality assumption. Tests were performed two-tailed. R-values were calculated via Spearman-Correlation. Log-rank (Mantel-Cox) test was used for comparison of survival curves. Significance levels: *p < 0.05; **p < 0.01; ***p < 0.001; ****p < 0.0001, Confidence Interval: 95%. Error bars in figures represent SD.

### Targeted sanger sequencing

Targeted Sanger sequencing of the region encompassing codon 409 of the human KLF4 gene was performed after PCR amplification. To monitor AKT1 mutations we also performed targeted Sanger sequencing of AKT1 (codon 17). For Primers and further details see supplementary materials.

### Tissue microarray (TMA)

Using 27 FFPE-embedded skull base WHO I° meningioma samples, a tissue microarray (TMA) was constructed as previously reported [25]. From each FFPE block, three separate samples were spotted on the TMA. Immunohistochemistry was performed from 4 μm thick paraffin sections. Antibodies used are detailed in supplementary materials.

### RNA-Seq and analysis of RNA-Seq data

#### Sample preparation and isolation of RNA for RNA sequencing

RNA was extracted from frozen tumor samples and sequenced by Novogene. (See supplementary materials for detailed protocol) RNA sequencing was performed on Illumina HiSeq X platform.

#### RNA sequencing data analysis

For RNA-seq data analyses low quality read ends as well as remaining parts of sequencing adapters were clipped off using Cutadapt (v 1.14). Subsequently, reads were aligned to the human genome (UCSC GRCh38) using HiSat2 (v 2.1.0) [15]. FeatureCounts (v 1.5.3) [20] was used for summarizing gene-mapped reads. Ensembl (GRCh38.89) [41] was used for annotating genes. Differentially expressed genes (DEGs) were determined by utilizing the R package edgeR (v 3.18.1) [30] using TMM normalization.

### Cell lines

The cell lines used were generated from the malignant meningioma cell line IOMM-Lee and cultured as previously described [25]. HEK293T cells were purchased from DSMZ (Braunschweig, Germany).

### Lentiviral transfection

HEK293T cells were lentivirally transfected using FuGene® HD transfection reagent (Promega, Mannheim, Germany) with pLV[Exp]-Bsd-EF1A > hKLF4[NM_004235.4] or pLV[Exp]-Bsd-EF1A > hKLF4[NM_004235.4]*(K409Q) constructs (VectorBuilder) in combination with lentiviral packaging plasmid mix pC-Pack 2 (Cellecta). After 48 h the supernatants were harvested, filtered and used to infect IOMM-Lee cells. Infected cells were finally selected with Blasticidin (Corning).

### Real-time reverse transcription (RT)-qPCR

RNA from adherent cells was isolated with TRIzol reagent (ThermoFischerScientific, Dreieich, Germany) or RNeasy Mini kit (QIAGEN) as described earlier [4]. Briefly, isolated RNA was reverse transcribed into cDNA using random hexamer primers and used for real-time PCR to determine the relative gene expression, normalized to a housekeeping gene (HPRT or β2-microglobulin). For genes analyzed, the primer sequences and systems used see supplementary materials. The relative mRNA expression was calculated through 1/2^∆Ct^, ∆Ct representing the difference of C_t_ values between gene of interest and housekeeping gene. For experiments involving hypoxic conditions Cells were cultured under hypoxia (1% O_2_) for 1 day before total RNA isolation.

### Cell proliferation

To determine cell proliferation, the Cell Proliferation ELISA (BrdU) from Roche was used. Ten thousand cells were seeded into 96-well plate (Sarstedt, Nümbrecht, Germany) and incubated at 37 °C, 5% CO2. Upon 48 h cells were stained according to manufacturer’s information and analyzed using Tecan-Reader (Infinite 200).

### Cell viability assay

The number of viable cells was determined by quantification of ATP in the cell culture using CellTiter-Glo® luminescent cell viability assay (Promega, Mannheim, Germany) according the manufacturer’s instruction. (Detailed protocol in Supplementary Materials).

### Colony formation assay

Ten thousand cells in 10 ml culture medium were seeded onto 10 cm culture dishes (TPP, Switzerland). After 4 days of cultivation, cells were washed with phosphate buffered saline (PBS; PAN Biotech, Aidenbach, Germany) and stained with 0,5% crystal violet solution containing 20% methanol for 15 min. Finally, the colony sizes were determined under a microscope with AxioVision Rel. 4.8 software.

### In vitro treatment of cells

0.1 × 10^6^ cells were seeded into cell culture flasks (25 cm^2^, Greiner bio-one) for gene expression studies, while 10,000 cells were seeded into 10 cm dishes for colony formation assays (CFA). Drugs were added after 24 h and remained on the cells for 24 h prior to protein or RNA extraction and 4 days prior to measuring of colonies in CFA. Stocks of Temsirolimus (Sigma) in ethanol and Simvastatin (Sigma) in DMSO were prepared. Final solvent concentrations after treatment was limited to 0.1% (vol/vol). Controls were treated with the solvent equivalent of the highest drug concentration.

### Immunoblotting

Cells or tissues were lysed in lysis buffer (see supplementary materials). In hypoxia experiments cells were treated with hypoxia (1% O_2_) or Temsirolimus (0.5 μM) for 24 h prior to lysis. Twenty micrograms of protein samples were separated on SDS polyacrylamide gels and transferred to a nitrocellulose membrane. Membranes were incubated with primary antibodies at 4 °C overnight and the protein expression was detected using horseradish peroxidase (HRP)-conjugated secondary antibodies and the chemiluminescent substrate. β-actin or Tubulin were used as loading controls. Blots were quantified using Kodak 1D3.6 software. (For antibodies and dilutions used see supplementary materials).

### Protein stability

HEK293T cells were transfected with GFP-KLF4-wt-pLVX or GFP-KLF4-mut(K409Q)-pLVX and selected with puromycin to generate a stable overexpression. Protein translation was blocked with Emetin [100 μM] at indicated time points and protein abundance was measured via immunoblot.

### Luciferase reporter assay

Cells were transiently transfected with constructs expressing firefly luciferase fused to HIF1α-ODD (Addgene plasmid #18965) together with an SV40-Renilla luciferase construct (Promega) used for normalization of transfection efficiency. Cells were cultured for 18 h under hypoxia and assayed for luciferase activity with the Dual-Luciferase Reporter-Assay System (Promega).

### Hydroxylation assay

2.5*10^5^ cells were cultured under hypoxia (1% O_2_) for 4 or 18 h. In the last 3 h before harvesting, cells were treated with MG132 (10 μM, Merck). Hydroxylation of HIF-1α on proline 564 was detected by blotting Hydroxy-HIF-1α (Pro564) antibody (Cell Signaling, 3434).

### Orthotopic xenograft mouse models

#### Convexity meningioma model

Young Swiss Nude mice (Charles River, France), > 9 weeks old were anesthetized intraperitoneally (i.p.) with Rompun (Bayer Vital GmbH Leverkusen, Germany) /Ketamin (Bremer Pharma GmbH, Warburg, Germany) mixture and fixed in the stereotactic head frame. After a longitudinal incision two holes were drilled 2 mm anterior of the bregma and 1.5 mm right and left from the sagittal suture. Using Hamilton syringe (Hamilton Bonaduz AG, Bonaduz, Switzerland) 2.5 × 10^5^ of KLF4^wt^ or KLF4^K40Q^ transfected IOMM-Lee meningioma cells in 2.5 μl PBS (PBS; PAN Biotech, Aidenbach, Germany) were applied 1.5 mm deep in each hole. The skin was sealed with Histoacryl (B Braun Surgical, S.A., Rubi, Spain).

#### Skull base meningioma model

Young Swiss Nude mice, > 9 weeks old, were used. A single hole was drilled 1.5 mm anterior of the bregma and 2 mm on the right side from the sagittal suture and 0.5 × 10^5^ transfected IOMM-Lee cells (*KLF*^wt^ or *KLFK*^409Q^) in 0.5 μl PBS were deposited above the skull base bone (about 7.5 mm in depth). At day three after tumor inoculation, daily treatment with Temsirolimus i.p. (20 mg/kg) was started for 11 days. At day 14 after tumor inoculation, tumor growth was analyzed by MRI. Tumor area was determined by tracing the largest tumor cross section on a given MRI slice.

#### Confocal microscopy

For actin staining 10,000 cells were grown in culture medium on non-treated cover slips at 37 °C, 5% CO2 overnight, fixed in 4% paraformaldehyde (PFA)/PBS for 10 min, permeabilized with 1% Triton X-100/PBS for 3–5 min and blocked with 1% BSA for 30 min. Actin was stained with Phalloidin Alexa Fluor 546 (Invitrogen, USA)/ 40x in PBS for 30 min. Cover slips were mounted on slides and imaged under a confocal microscope TCSSL (Leica, Germany).

#### Study approval

Study of human tumor specimens was performed after written consent was received from individual patients. The study was approved by the local ethics board of University Hospital of Cologne, (Application No. 03–170). Animal studies were approved by the local state department: Landesverwaltungsamt Sachsen Anhalt, Referat Verbraucherschutz, Veterinärangelegenheiten; Licence number 42502–2-1550 UniMD.

## Results

### KLF4^K409Q^ mutation is associated with a significant upregulation of hypoxia driven pathways

**Fig. 1:**
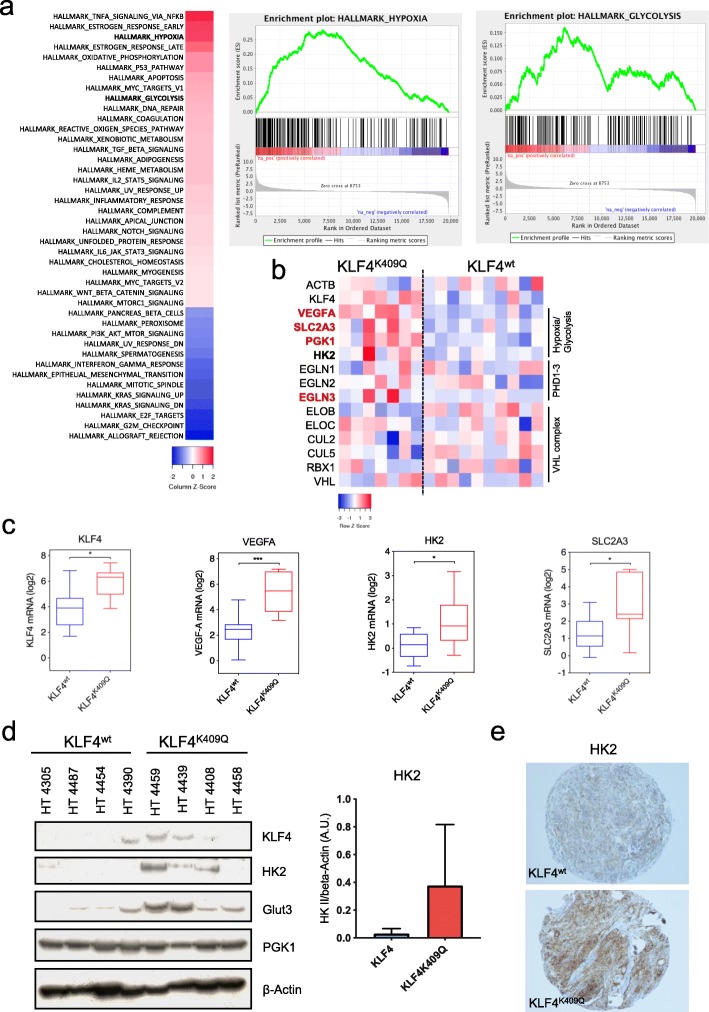
KLF4^K409Q^ Mutation results in a significant shift of gene expression and upregulates hypoxia driven pathways. **a** Transcriptomic analysis of 7 *KLF4*^*K409Q*^ and 10 KLF4^wt^ tumors. Nineteen thousand seven hundred ninety-nine protein-coding transcripts were considered, Trimmed Mean of M-value-normalization of all data. False discovery rate (FDR) < 0.1. Heatmap indicates the NES values derived for indicated hallmark gene sets by GSEA. Only gene sets with an FDR < 0.05 were considered. **b** Selective heatmap of genes relevant to hypoxia/glycolysis pathways. Red indicates significantly (FDR < 0.05) upregulated genes in KLF4^K409Q^ tumors, bold is upregulated but not significant. **c** Boxplots of *KLF4, VEGF-A, SLC2A3* and *HK2* expression within the analyzed samples. **d, e** Exemplary Western blot, its quantification of HK2 and TMA-IHC (**e**) of patient derived tumor-tissue confirming the increased expression of hypoxia dependent genes as well as KLF4 on the protein level in *KLF*^*K409Q*^ Meningiomas. (Western blot: *n* = 8, 4 KLF4^wt^ and 4 KLF4K409Q, TMA: *n* = 27, 17 KLF4^wt^ and 10 KLF4^K409Q^)

Clinical and pathological data on 96 patients who previously underwent meningioma resection between 2013 and 2018 were collected and frozen tumor samples sequenced for the *KLF4*^*K409Q*^ mutation. Of the 96 meningiomas, 81 (84,4%) were WHO I°, 14 (14.6%) WHO II° and one (1,04%) was WHO III°. 13 (13.5%) of the analyzed tumors carried the *KLF*^*K409Q*^mutation and were significantly associated with a secretory subtype (*p* < 0.001) and sphenoid wing location (*p* < 0.005), confirming previous reported observations [5, 29]. In order to gauge the impact of the KLF4K409Q mutation on cellular pathways, we performed transcriptomic analysis of 7 *KLF4*^*K409Q*^ and 10 *KLF4*^wt^ meningiomas (all WHO I° and *AKT1* wildtype) matched by patient sex, age and tumor location. Unsupervised clustering revealed a significant shift in gene expression of mutated tumors with strong upregulation of hypoxia-induced pathways. These findings were supported by Gene-set-enrichment analysis (GSEA) pointing at upregulation of hypoxia-inducible factor (HIF-1α)-dependent gene expression (Fig. 1a). Next to an overall increased KLF4 level, direct comparison of mRNA levels, tissue-micro-array (TMA) staining and Western blot analysis confirmed the increase of HIF-1α dependent genes such as Hexokinase II and SLC2A3 in *KLF4*^*K409Q*^ tumors. Significant upregulation of PGK1 mRNA did not translate to increased PGK1 protein levels in mutated samples. (Fig. 1b, c, d, e). Given the fact that KLF4K409Q mutated tumors are strongly associated with PTBE and elevated VEGF levels are characteristic for meningiomas with large PTBE [24, 37], we aimed to further investigate the involvement of the *KLF4*^*K409Q*^-mutation on the regulation of VEGF and more generally on the hypoxia pathway experimentally.

### The KLF4^K409Q^-mutation reduces growth rate and leaves cells susceptible to hypoxia

**Fig. 2:**
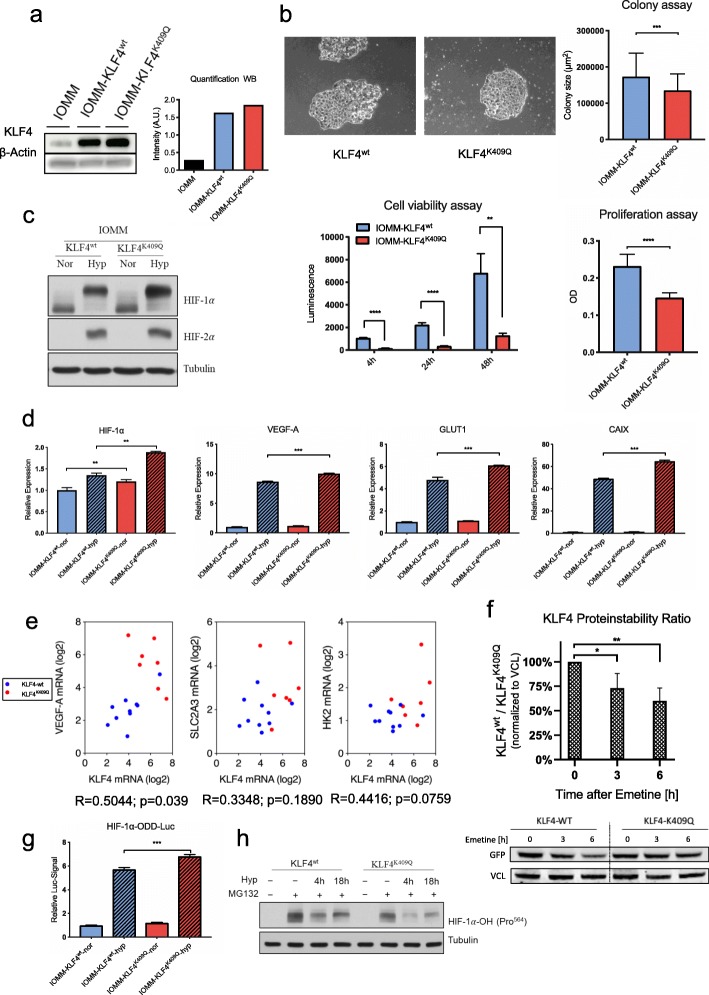
Effects of the KLF4^K409Q^ mutation on cell viability, growth rate and hypoxia response. **a** Western blot analysis of KLF4 overexpression in transfected IOMM cells. **b** Colony formation assay (*n* = 60), cell viability assay after 4, 24 and 48 h (*n* = 5 / timepoint) and cell proliferation assay (*n* = 15) of KLF4^wt^- and KLF4^K409Q^ -cell lines. **c, d** Western Blot and RT-qPCR (*n* = 3) analyses of cell lines under normoxic and hypoxic conditions. **e** Correlation of KLF4-mRNA and mRNA levels of hypoxia dependent genes in patient derived tumor samples. **f** Quantification of relative KLF4^wt^/^K409Q^ protein stability with GFP-KLF4 constructs and blocking of protein translation with emetin at specified timepoints. (*n* = 3). **g** Quantification of hydroxylation dependent HIF1-α degradation in IOMM-*KLF4*^wt^/^K409Q^ cell lines after transient transfection of *HIF1-ODD-Luc* (*n* = 3). **h** Western Blot analysis of hydroxylated HIF1-α after proteasome inhibition with MG132

To this end, we generated 2 cell lines from IOMM-Lee cells, an immortal, NF2 wildtype line of meningothelial tumor cells [18], with a lentivirus carrying *KLF4*^*wt*^ or *KLF4*^*K409Q*^ with a *BSD*-tag (*BSD* selectable marker) under the control of an *EF1-α* promoter. Stable cell lines were selected with Blasticidin and comparable levels of KLF4 overexpression in both *KLF4*^*wt*^ and *KLF4*^*K409Q*^ cells were confirmed by western blotting (Fig. 2a). Comparison of cell proliferation, cell viability and colony formation capacities of the cell lines revealed that the *KLF4*^*K409Q*^ meningioma cells had a significantly less aggressive character than the *KLF4*^wt^ cells, in line with the smaller tumor size associated with *KLF4*^*K409Q*^ meningiomas [43] (Fig. 2b). RT-qPCR analysis of HIF-1α-dependent gene expression revealed a consistent upregulation of target gene mRNA levels in the IOMM-*KLF4*^*K409Q*^ cells (Fig. S1). Given the fact that the hypoxic pathway is upregulated in the patient derived tumor samples, we assessed the effect of *KLF4*^*K409Q*^ on the hypoxia pathway in IOMM cells under hypoxic conditions. Cells grown for 24 h under normoxic (control) or hypoxic conditions were harvested and relative mRNA expression as well as protein levels analyzed. Importantly, *KLF4*^*K409Q*^ lead to a robust increase of HIF-1*α* (and to a lesser extent of HIF-2*α*) under normoxic and hypoxic conditions. In line with a HIF-dependent induction of the hypoxia pathway through *KLF4*^*K409Q*^ the upregulation of the HIF-target genes *GLUT1, VEGF and CAIX* in *KLF4*^*K409Q*^ cells was significantly larger (*p* < 0.001) when compared to *KLF4*^*wt*^ cells under hypoxic conditions (Fig. 2c, d).

### The KLF4^K409Q^ mutation promotes KLF4 protein stability and inhibits hydroxylation-dependent degradation of HIF1-α

**Fig. 3:**
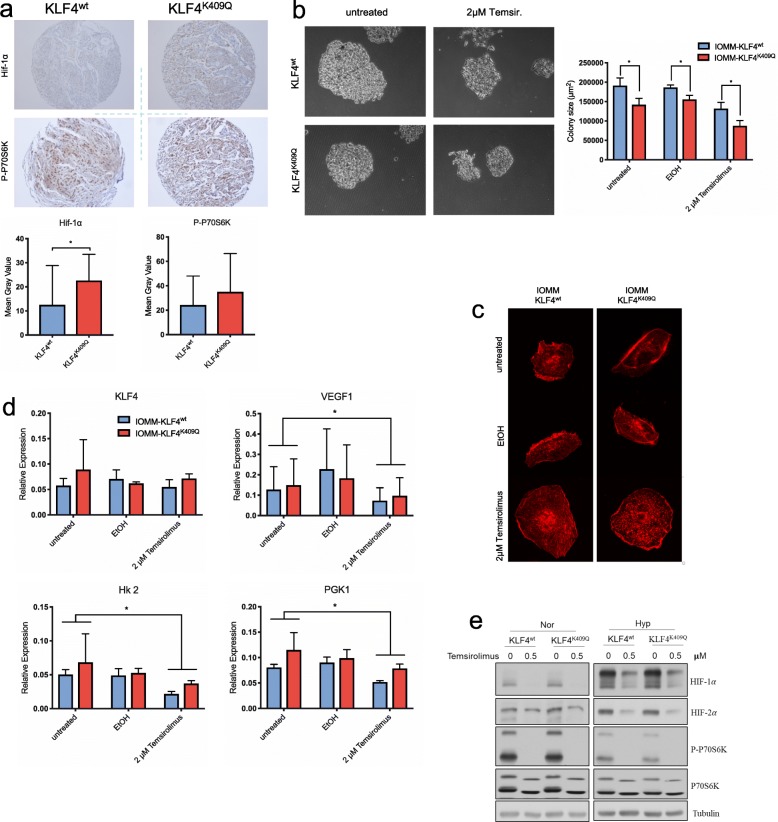
Effect of Temsirolimus treatment in vitro. **a** TMA staining of human tumor samples for HIF1-α and Pho-p70S6k. (*n* = 27, 17 KLF4^wt^, 10 KLF4^K409Q^). **b** Colony formation assay (CFA) and size analysis after Temsirolimus treatment. (*n* = 60). **c** Confocal imaging of actin stained IOMM cells. **d** RT-qPCR analysis of HIF1-α dependent genes after treatment with Temsirolimus under normoxic conditions. (*n* = 3). **e** Western Blot analysis of HIF1-α and (Ph)-p70S6K protein levels after treatment with Temsirolimus under hypoxia

Our initial hypothesis was that increased expression of KLF4 would direct the upregulation of hypoxic response genes in mutated meningiomas. However, the correlation of KLF4 mRNA levels with the mRNA levels of *VEGFA, SLC2A3* and *HK2* was only moderate to weak in the analyzed tumor samples (*R* = 0.5044; *R* = 0.3348; *R* = 0.4416; Fig. 2e). Thus, upregulation of KLF4 mRNA is unlikely to be the sole reason for enhanced hypoxia and glycolysis, which was detected by GSEA. Therefore, we investigated if the *KLF4*^*K409Q*^ mutation could affect the protein stability of KLF4. To this end, HEK293T cells were transfected with *GFP-KLF4*^*wt*^ or -^*K409Q*^ construct and selected for stable expression. After inhibiting protein translation with Emetin, GFP-KLF4 levels were measured at 0, 3 and 6 h. After both 3- and 6-h blockage, GFP-KLF4 levels were significantly higher (*p* < 0.05; *p* < 0.005) in the KLF4^*K409Q*^ group, suggesting that increased protein stability contributes to the elevated KLF4 activity in these cells (Fig. 2f). However, the mechanisms underlying the striking difference between the hypoxia response of *KLF4*^*wt*^ cells and *KLF4*^*K409Q*^ cells (Fig. 2c, d) remained unexplained. We therefore further investigated the effect of the *KLF4*^*K409Q*^ mutation on HIF-1α. Since HIF-1α is characterized by a high protein turnover regulated by oxygen-dependent degradation, we tested whether the *KLF4*^*K409Q*^ mutation affects this process. We co-transfected both KLF4 cell lines with a *HIF-1α-ODD-Luciferase* [32] which transfers the oxygen-dependent sensitivity of HIF-1α to firefly luciferase*,* and a Renilla-Luciferase control construct and quantified the bioluminescence under hypoxic conditions. Importantly, *KLF4*^*K409Q*^ significantly increased the stability of the ODD reporter construct, showing that *KLF4*^*K409Q*^ reduces the oxygen-dependent degradation of HIF-1α (Fig. 2g). This was further confirmed by assessing the prolyl hydroxylation of HIF-1α. Hydroxylation of proline residues in HIF-1α ODD-domain is required for pVHL binding which leads to HIF-1α ubiquitination and degradation. This process is catalyzed by different oxygen-dependent prolyl-4-hydroxylases (PHDs) [21]. To analyze PHD activity, we subjected KLF4 cells to hypoxia, inhibited the proteasome activity with MG132 and analyzed protein levels of hydroxylated HIF-1α (HIF-1α-OH) after 0, 4 and 18 h. Consistently, HIF-1α-OH levels in *KLF4*^*K409Q*^ cells were prominently reduced at all timepoints (0, 4 and 18 h after treatment) (Fig. 2h). Importantly, TMA-staining of the matched *KLF4*^wt^ / *KLF4*^*K409Q*^ meningioma samples confirmed the increased expression of HIF-1α in *KLF4*^*K409Q*^ meningiomas (Fig. 3a)*.* Collectively, these experiments demonstrate that KLF4^K409Q^ potentiates HIF-1α and HIF dependent gene expression through the reduction of PHD activity.

### KLF4^K409Q^ tumors show susceptibility to mTORC1 inhibition

The search of public databases for compound candidates that directly inhibit KLF4, revealed statins as a promising option. They are a well-established class of drugs with a good safety profile which have been shown to be cytotoxic against meningioma cells [10] and were recently reported to act as specific inhibitors of KLF4 in cancer stem cells [19]. Unfortunately, the effect of Simvastatin (a medication in Statins class) did not translate into our KLF4 meningioma model (Fig. S2).

Given the fact that the *KLF4*^*K409Q*^ mutation leads to specific upregulation of the hypoxia pathway, we explored whether this could be exploited for medical treatment. We have shown previously that the mTOR (mammalian target of rapamycin) inhibitor Temsirolimus is a promising agent in meningioma treatment [25]. Furthermore, mTOR inhibitors have proven effective in the treatment of clear renal cell carcinoma, a cancer associated with loss of pVHL function and deregulation of hypoxia pathways [2]. In general, HIF-1α levels are increased following mTOR activation through phosphorylation of both the eukaryotic translation initiation factor 4E binding protein (4E-BP1) and the p70-S6 kinase (p70S6K) [21]. Since TMA-staining of the matched *KLF4*^wt^ / *KLF4*^*K409Q*^ meningioma samples revealed a trend towards higher expression of both p70S6K and phosphorylated p70S6K (Pho-p70S6K) in the mutated samples (Fig. 3a; Fig. S3), we decided to test the effect of Temsirolimus on cell growth in vitro.

Cells treated with Temsirolimus formed significantly smaller colonies, had strongly altered morphology (Fig. 3b, c) and KLF4 as well as its dependent genes VEGF, HK2 and PGK1 were downregulated (Fig. 3d). Importantly, we were able to confirm that this effect was equally strong under hypoxic conditions. Treatment with Temsirolimus reliably reduced the overshooting HIF-1α expression in both cell lines under hypoxia (Fig. 3e).

#### Temsirolimus is a promising treatment option for meningiomas in vivo

**Fig. 4:**
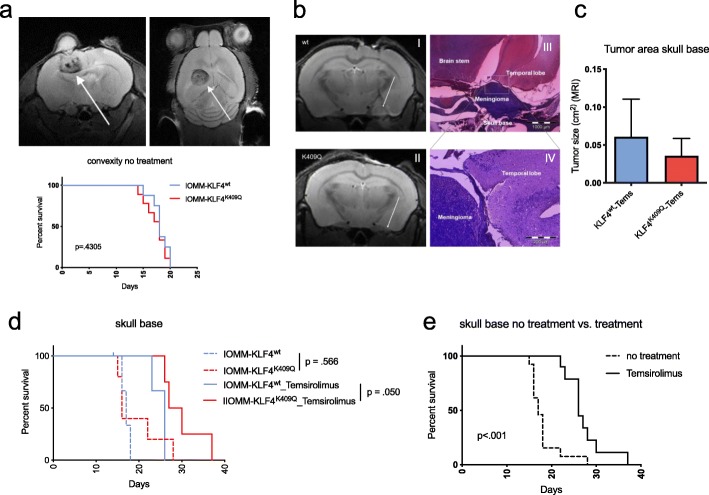
Survival studies of orthotopic xenograft models in mice. **a** Kaplan-Meier-Survival curve after establishing tumors through intracranial injection of IOMM-*KLF4*^wt^ or -*KLF4*^K409Q^ cells in the convexity (IOMM-*KLF4*^*wt*^ vs IOMM-*KLF4*^*K409Q*^) and exemplary MRI. No difference in OS was observed (*p* = .4305). **b** Exemplary MRI of KLF4^wt^ (I) and KLF4^K409Q^ (II) tumors and histological cross section of the *KLF4*^*K409Q*^ skull base tumor (III and IV). **c** Analysis of tumor size on MRI (*n* = 3). **d** Kaplan-Meier-Survival curve of mice bearing skull base tumors with and without treatement. **e** Combined Kaplan-Meier-Survival curve of both cell lines (KLF4^wt^ and KLF4^K409Q)^ with and without treatment

To evaluate our findings in vivo we first performed xenograft experiments by establishing convexity tumors through intracranial injection of IOMM-*KLF4*^wt^ or -*KLF4*^K409Q^ cells. The results show no difference in tumor formation or overall survival (OS) for tumors grown at this meningioma site (Fig. 4a). Subsequently, to better mimic clinical conditions of skull-base meningiomas, we implanted tumor cells at the skull base and treated mice with the mTOR inhibitor Temsirolimus. While not being statistically significant, MRI of tumor-bearing mice showed that *KLF4*^K409Q^ tumors at the skull base tended to be smaller than their wildtype counterpart, replicating clinical findings of small tumor sizes in *KLF4*^K409Q^ meningioma (Fig. 4b, c). When treated with Temsirolimus in the skull base meningioma model, median OS in both groups (*KLF4*^*wt*^ and *KLF4*^*K409Q*)^ was prolonged by 9 days (17 vs 26 days, *p* < 0.001) in comparison with non-treated mice. When comparing treated mice bearing skull base KLF4^wt^ or skull base KLF4^K409Q^ tumors, the median survival of the KLF4^K409Q^ group was further increased by 2.5 days (26 vs 28.5 days, *p*= 0.050) (Fig. 4d, e). Taken together, these data identify Temsirolimus as a promising agent preferentially in *KLF4*^K409Q^ skull base tumors.

## Discussion

Recent next generation sequencing efforts have led to a better understanding of the molecular drivers of meningioma. The *KLF4*^*K409Q*^ mutation defines a distinct subtype of meningioma characterized by a preferential skull base location, large edema formation and slow growth [5, 29, 43]. Here we identify a crucial role of the *KLF4*^*K409Q*^ mutation in the control of the hypoxic pathway through HIF-1α and uncover novel therapeutic options to specifically treat *KLF4*^*K409Q*^ mutated meningiomas.

The first line of treatment for a small, asymptomatic meningioma usually consists of a simple “watch and wait” approach [11]. Since *KLF4*^*K409Q*^ mutated tumors frequently show slow tumor growth this would seemingly be a logical first step. However, *KLF4*^*K409Q*^ mutated tumors are prone to cause severe PTBE, which in turn tends to cause a variety of severe neurological symptoms and in most cases can only be alleviated by surgical tumor resection. Due to the challenging skull base location [7], the increased age of meningioma patients [26], and heightened risks of serious periprocedural complications in tumors with large PTBE [28], the need for alternative treatment options is obvious.

Our data uncover that the increased activation of the HIF pathway is a consequence of the *KLF4*^*K409Q*^ mutation in meningioma which could contribute to several biological characteristics of this tumor subtype. Our transcriptomic analysis revealed the upregulation of the hypoxia pathway in *KLF4*^*K409Q*^ tumors. This observation was recapitulated in vitro in IOMM-Lee cells overexpressing KLF4^K409Q^ strongly suggesting a functional role for the *K409Q*-mutation as the main driver of this change. We show that the *KLF4*^*K409Q*^ leads to increased activation of HIF-1α under normoxia and hypoxia. Mechanistically, we demonstrate that the KLF4^*K409Q*^ mutation reduces oxygen-dependent HIF1-α degradation through decreased hydroxylation.

HIF-1α has been characterized as one of the main drivers of VEGF expression in tumors and increased levels of VEGF have long been thought to play a crucial role in the formation of PTBE. However, until now the underlying factors leading to increased VEGF expression in the context of meningiomas have remained elusive.

The activation of the HIF pathway in solid tumors is a common phenomenon and has been linked to high proliferation, angiogenesis and poor prognosis in different cancers [16, 17, 40]. Importantly, we and others have previously demonstrated that the HIF pathway also entails tumor-suppressive components and can reduce tumor proliferation and growth [1, 22], possibly explaining the benign properties of KLF4^K409Q^ mutated tumors. Prototype tumor mutational changes of PI3K, p53 and pVHL in several cancers have been shown to converge on the HIF pathway, directly or indirectly altering HIF’s translation, its degradation and the activity of its downstream targets [14].

Similarly, we show that in meningiomas the *KLF4*^*K409Q*^ mutation results in an increased HIF-1α activity through impaired HIF-1α degradation. Our experiments show that the *KLF4*^*K409Q*^ mutation leads to an increased stability of KLF4. Since both HIF-1α and KLF4 are degraded after ubiquitinoylation through the pVHL complex [9], increased KLF4 levels could lead to the competitive binding of pVHL and reduce ubiquitinoylation of HIF-1α. Additionally, *KLF4*^*K409Q*^ reduces oxygen dependent hydroxylation of HIF1-α and further decreases degradation.

Anti-edematous therapy in neuro-oncology has relied on dexamethasone for decades. While the efficacy of this treatment is well established, side effects for long term users are severe. In our study we show that by leveraging the mTor dependency of HIF1-α, Temsirolimus suppresses the overshooting hypoxia response, reduces VEGF levels under normoxic and hypoxic conditions and inhibits tumor growth both in vitro and in vivo in KLF4-overexpressing tumor cells. In *KLF4*^*K409Q*^ mutated cells this effect is even more pronounced than in *KLF4*^*wt*^. Given the strong link between HIF-1α and VEGF expression, we postulate that this would translate into relevant reductions of PTBE. However, due the general lack of a viable PTBE mouse model, this remains hypothetical. Apart from Temsirolimus, direct VEGF inhibitors like bevacizumab might prove useful in clinical treatment in the subgroup of meningiomas exhibiting a derailed HIF pathway.

## Conclusion

In summary, this study provides important mechanistic insights into the biological characteristics of *KLF4*^*K409Q*^ mutated meningiomas and could provide a starting point for alternative treatment options in patients with skull base meningiomas.

## Supplementary information

**Additional file 1: Supplementary Materials and Methods: Figure S1.** RT-qPCR analysis of HIF-1α-dependent genes in KLF4^wt/K409Q^ transfected cells (*n* = 4). **Figure S2.** Evaluation of Simvastatin treatment in IOMM-KLF4 cell lines. **Figure S3.** TMA-staining for p70S6K and CD31 (*n* = 23).

## References

[1] Acker T, Diez-Juan A, Aragones J, Tjwa M, Brusselmans K, Moons L, Fukumura D, Moreno-Murciano MP, Herbert JM, Burger A (2005). Genetic evidence for a tumor suppressor role of HIF-2alpha. Cancer Cell.

[2] Battelli C, Cho DC (2011). mTOR inhibitors in renal cell carcinoma. Therapy.

[3] Boetto J, Bielle F, Sanson M, Peyre M, Kalamarides M (2017). SMO mutation status defines a distinct and frequent molecular subgroup in olfactory groove meningiomas. Neuro-Oncology.

[4] Chomczynski P, Sacchi N (1987) The single-step method of RNA isolation by acid guanidinium thiocyanate–phenol–chloroform extraction: twenty-something years on. Nat Protoc. 1(2):581–585 10.1038/nprot.2006.83 17406285

[5] Clark VE, Erson-Omay EZ, Serin A, Yin J, Cotney J, Ozduman K, Avşar T, Li J, Murray PB, Henegariu O (2013). Genomic analysis of non-NF2 meningiomas reveals mutations in TRAF7, KLF4, AKT1, and SMO. Science (New York, NY).

[6] Cordes KR, Sheehy NT, White MP, Berry EC, Morton SU, Muth AN, Lee TH, Miano JM, Ivey KN, Srivastava D (2009). MiR-145 and miR-143 regulate smooth muscle cell fate and plasticity. Nature.

[7] Cornelius JF, Slotty PJ, Steiger HJ, Hänggi D, Polivka M, BJAn G (2013). Malignant potential of skull base versus non-skull base meningiomas: clinical series of 1,663 cases. Acta Neurochir.

[8] Evans PM, Liu C (2008) Roles of Krüppel‐like factor 4 in normal homeostasis, cancer and stem cells. Acta Biochimica et Biophysica Sinica 40(7):554–64. 10.1111/j.1745-7270.2008.00439.x PMC2668950 18604447

[9] Gamper AM, Qiao X, Kim J, Zhang L, DeSimone MC, Rathmell WK, YJMc W (2012). Regulation of KLF4 turnover reveals an unexpected tissue-specific role of pVHL in tumorigenesis. Mol Cell.

[10] Gehring S, Tapia-Pérez JH, Kirches E, Firsching R, Keilhoff G, Schneider T, Mawrin C (2011) Cytotoxic effects of statins and thiazolidinediones on meningioma cells. J Neurooncol 102(3):383–93. 10.1007/s11060-010-0351-1 20803306

[11] Goldbrunner R, Minniti G, Preusser M, Jenkinson MD, Sallabanda K, Houdart E, von Deimling A, Stavrinou P, Lefranc F, Lund-Johansen M (2016). EANO guidelines for the diagnosis and treatment of meningiomas. Lancet Oncol.

[12] Harsha B, Creatore C, Kok CY, Hathaway C, Cole CG, Ramshaw CC, Rye CE, Beare DM, Dawson E, Boutselakis H (2018). COSMIC: the catalogue of somatic mutations in cancer. Nucleic Acids Res.

[13] Karlsson B, Kihlström L, Lindquist C, Mathiesen T (1996). Recurrence of Cranial Base Meningiomas. Neurosurgery.

[14] Keith B, Johnson RS, Simon MC (2012) HIF1α and HIF2α: sibling rivalry in hypoxic tumour growth and progression. Nat Rev Cancer 12(1):9–22. 10.1038/nrc3183 PMC3401912 22169972

[15] Kim D, Langmead B, Salzberg SL (2015). HISAT: a fast spliced aligner with low memory requirements. Nat Methods.

[16] Klatte T, Seligson DB, Riggs SB, Leppert JT, Berkman MK, Kleid MD, Belldegrun AS (2007) Hypoxia-inducible factor 1α in clear cell renal cell carcinoma. Clin Cancer Res 13(24):7388–393. 10.1158/1078-0432.CCR-07-0411 18094421

[17] Korkolopoulou P, Patsouris E, Konstantinidou AE, Pavlopoulos PM, KavantzasN, BoviatsisE, Rologis D (2004) Hypoxia‐inducible factor 1α/vascular endothelial growth factor axis in astrocytomas. Associations with microvessel morphometry, proliferation and prognosis. Neuropathol Appl Neurobiol 30(3):267–78. 10.1111/j.1365-2990.2003.00535.x 15175080

[18] Lee WH (1990). Characterization of a newly established malignant meningioma cell line of the human brain: IOMM-Lee. Neurosurgery.

[19] Li Y, Xian M, Yang B, Ying M, He Q (2017). Inhibition of KLF4 by statins reverses adriamycin-induced metastasis and cancer stemness in osteosarcoma cells. Stem Cell Rep.

[20] Liao Y, Smyth GK, Shi W (2014). featureCounts: an efficient general purpose program for assigning sequence reads to genomic features. Bioinformatics (Oxford, England).

[21] Masoud GN, Li W (2015). HIF-1α pathway: role, regulation and intervention for cancer therapy. Acta Pharm Sin B.

[22] Meléndez-Rodríguez F, Urrutia AA, Lorendeau D, Rinaldi G, Roche O, Böğürcü-Seidel N, Hernansanz-Agustín P (2019) HIF1α suppresses tumor cell proliferation through inhibition of aspartate biosynthesis. Cell Rep 26(9):2257–265. 10.1016/j.celrep.2019.01.106 30811976

[23] Ostrom QT, Gittleman H, Liao P, Vecchione-Koval T, Wolinsky Y, Kruchko C, Barnholtz-Sloan JS (2017). CBTRUS statistical report: primary brain and other central nervous system tumors diagnosed in the United States in 2010–2014. Neuro-Oncology.

[24] Otsuka S, Tamiya T, Ono Y, Michiue H, Kurozumi K, Daido S, Kambara H, Date I, Ohmoto T (2004). The relationship between peritumoral brain edema and the expression of vascular endothelial growth factor and its receptors in intracranial meningiomas. J Neuro-Oncol.

[25] Pachow D, Andrae N, Kliese N, Angenstein F, Stork O, Wilisch-Neumann A, Kirches E, Mawrin C (2013). mTORC1 inhibitors suppress meningioma growth in mouse models. Clin Cancer Res.

[26] Patil CG, Veeravagu A, Lad SP, Boakye M (2010). Craniotomy for resection of meningioma in the elderly: a multicentre, prospective analysis from the National Surgical Quality Improvement Program. J Neurol Neurosurg Psychiatry.

[27] Preusser M, Brastianos PK, Mawrin C (2018). Advances in meningioma genetics: novel therapeutic opportunities. Nat Rev Neurol.

[28] Regelsberger J, Hagel C, Emami P, Ries T, Heese O, Westphal M (2009). Secretory meningiomas: a benign subgroup causing life-threatening complications. Neuro-Oncology.

[29] Reuss DE, Piro RM, Jones DTW, Simon M, Ketter R, Kool M, Becker A, Sahm F, Pusch S, Meyer J (2013). Secretory meningiomas are defined by combined KLF4 K409Q and TRAF7 mutations. Acta Neuropathol.

[30] Robinson MD, McCarthy DJ, Smyth GK (2010). edgeR: a bioconductor package for differential expression analysis of digital gene expression data. Bioinformatics (Oxford, England).

[31] Rowland BD, Bernards R, Peeper DS (2005). The KLF4 tumour suppressor is a transcriptional repressor of p53 that acts as a context-dependent oncogene. Nat Cell Biol.

[32] Safran M, Kim WY, O'Connell F, Flippin L, Gunzler V, Horner JW, Depinho RA, Kaelin WG (2006). Mouse model for noninvasive imaging of HIF prolyl hydroxylase activity: assessment of an oral agent that stimulates erythropoietin production. Proc Natl Acad Sci U S A.

[33] Segre JA, Bauer C, Fuchs E (1999). Klf4 is a transcription factor required for establishing the barrier function of the skin. Nat Genet.

[34] Shields JM, Christy RJ, Yang VWJJBC (1996). Identification and characterization of a gene encoding a gut-enriched Krüppel-like factor expressed during growth arrest. J Biol Chem.

[35] Takahashi K, Tanabe K, Ohnuki M, Narita M, Ichisaka T, Tomoda K, Yamanaka S (2007). Induction of pluripotent stem cells from adult human fibroblasts by defined factors. Cell.

[36] Tang HL, Zhu HD, Wang XC, Hua LY, Li JR, Xie Q, Chen XC, Zhang T, Gong Y (2017). KLF4 is a tumor suppressor in anaplastic meningioma stem-like cells and human meningiomas. J Mol Cell Biol.

[37] Wang P, Ni RY, Chen MN, Mou KJ, Mao Q, Liu YH (2011). Expression of aquaporin-4 in human supratentorial meningiomas with peritumoral brain edema and correlation of VEGF with edema formation. Genet Mol Res.

[38] Wang Y, Yang C, Gu Q, Sims M, Gu W, Pfeffer LM, Yue J (2015). KLF4 promotes angiogenesis by activating VEGF signaling in human retinal microvascular endothelial cells. PLoS One.

[39] Wei D, Xie K, Kanai M, Huang S (2005). Emerging role of KLF4 in human gastrointestinal cancer. Carcinogenesis.

[40] Yamamoto Y, Ibusuki M, Okumura Y, Kawasoe T, Kai K, Iyama K, HJBcr I (2008) Hypoxia-inducible factor 1α is closely linked to an aggressive phenotype in breast cancer. 110:465–475 10.1007/s10549-007-9742-1 17805961

[41] Yamamoto Y, Ibusuki M, Okumura Y, Kawasoe T, Kai K, Iyama K, Iwase H (2008). Hypoxia-inducible factor 1α is closely linked to an aggressive phenotype in breast cancer. Breast Cancer Res Treat, 110(3):465–75. 10.1007/s10549-007-9742-1 17805961

[42] Yesiloz U, Kirches E, Hartmann C, Scholz J, Kropf S, Sahm F, Nakamura M, Mawrin C (2017) Frequent AKT1E17K mutations in skull base meningiomas are associated with mTOR and ERK1/2 activation and reduced time to tumor recurrence. Neuro-Oncology 19:1088–1096. 10.1093/neuonc/nox018 10.1093/neuonc/nox018 PMC5570238 28482067

[43] Youngblood MW, Duran D, Montejo JD, Li C, Omay SB, Özduman K, Sheth AH, Zhao AY, Tyrtova E, Miyagishima DF et al (2019) Correlations between genomic subgroup and clinical features in a cohort of more than 3000 meningiomas. J Neurosurg:1–10. 10.3171/2019.8.jns191266 10.3171/2019.8.JNS191266 31653806

[44] Yu F, Li J, Chen H, Fu J, Ray S, Huang S, Zheng H, Ai W (2011). Kruppel-like factor 4 (KLF4) is required for maintenance of breast cancer stem cells and for cell migration and invasion. Oncogene.

