# Supplementary material for: KLF4K409Q–mutated meningiomas show enhanced hypoxia signaling and respond to mTORC1 inhibitor treatment
Source: Acta Neuropathol Commun. 2020 Apr 3;8:41. doi: 10.1186/s40478-020-00912-x (PMC7118946; doi:10.1186/s40478-020-00912-x)
Supplement: Supplementary file 1 — Additional file 1: Supplementary Materials and Methods: Figure S1. RT-qPCR analysis of HIF-1α-dependent genes in KLF4wt/K409Q transfected cells (n = 4). Figure S2. Evaluation of Simvastatin treatment in IOMM-KLF4 cell lines. Figure S3. TMA-staining for p70S6K and CD31 (n = 23). [file 40478_2020_912_MOESM1_ESM.docx]

Title: *KLF4^K409Q^*–mutated meningiomas show enhanced hypoxia signaling and respond to mTORC1 inhibitor treatment

**Authors:** Niklas von Spreckelsen^1,2,3, †^, Natalie Waldt^1†^, Rebecca Poetschke^4†^, Christoph Kesseler^1^, Hildegard Dohmen^7^, Hui-Ke Jiao^7^, Attila Nemeth^7^,Stefan Schob^9^, Cordula Scherlach^9^, Ibrahim Erol Sandalcioglu^10^, Martina Deckert^5^, Frank Angenstein^6^, Boris Krischek^3^, Pantelis Stavrinou^3^, Marco Timmer^3^, Marc Remke^8^, Elmar Kirches^1^, Roland Goldbrunner^3^, E. Antonio Chiocca^2^, Stefan Huettelmaier^4^, Till Acker^7^, and Christian Mawrin^1*^

**Affiliations:**

^1^Department of Neuropathology, Otto-von-Guericke University, Magdeburg; Germany.

^2^Department of Neurosurgery, Brigham and Women’s Hospital, Harvard Medical School, U.S.A.

^3^Department of Neurosurgery, Center for Neurosurgery, Faculty of Medicine and University Hospital, University of Cologne, Cologne, Germany.

^4^Institute of Molecular Medicine, Martin Luther University, Halle/Saale, Germany.

^5^Department of Neuropathology, University Hospital Cologne, Germany.

^6^Laboratory for Non-invasive Imaging, Magdeburg, Germany.

^7^Department of Neuropathology, University Giessen, Germany.

^8^Pediatric Neuro-Oncology, Duesseldorf, Germany.

^9^Department of Neuroradiology, University Hospital Leipzig.

^10^Department of Neurosurgery, Otto-von-Guericke University, Magdeburg; Germany

* Correspondence to:

Christian Mawrin, MD

Department of Neuropathology

Otto-von-Guericke University Magdeburg, Germany

Tel: +49 391 6715825

Fax: +49 391 6713300

e-mail: [christian.mawrin@med.ovgu.de](mailto:christian.mawrin@med.ovgu.de)

^†^co-first authors

**Supplementary Materials and Methods:**

*Targeted Sanger Sequencing*

Sequencing was performed on an ABI-Prism-310C capillary sequencer (Applied Biosystems, Foster City, CA, USA), using Big-Dye-Terminator technology (Applied Biosystems).

Primers:

KLF4 forward: GCTCATGCCACCCGGTTC

reverse: CTGTGTGGGTTCGCAGGTG

AKT1 forward: CTGGCCCTAAGAAACAGCTCC

reverse: CGCCACAGAGAAGTTGTTGA

*Sample preparation and isolation of RNA for RNA sequencing*

Frozen tumor samples were shredded in liquid nitrogen with a mortar and allotted into 1.5ml Eppendorf tubes. For isolation of tissue RNA, ceramic beads were added to the frozen tissue pulver and supplemented with 1ml TRIzol. The vessel content was homogenized for 20 seconds with Precellys® 24 and stored for 5min on ice. RNA was isolated via TRIzol/Chloroform extraction. Total RNA Sequencing was performed by Novogene. NEB Next® Ultra™ RNA Library Prep Kit was used for RNA library preparation.

*Real-time reverse transcription (RT)-qPCR*

The RT reaction was performed by using Maxima H Minus First Strand cDNA synthesis Kit (Thermo Scientific). 1µg total RNA was incubated with oligo primer, random primer and dNTPs at 65 ºC for 5 minutes. RT buffer and Maxima H Minus enzyme were added and reaction was incubated at 25 ºC for 10 minutes followed by incubation at 50 ºC for 30 minutes and then inactivation of the enzyme by heating at 85 ºC for 5 minutes. The cDNAs were amplified using the primers listed below. PowerUp SYBR Green Master mix (Applied Biosystems) and a thermal cycler Quantstudio3 (Applied Biosystems). The reaction conditions were initial denaturation at 95 ºC for 2 minutes, 45 cycles of denaturation for 30s at 95 ºC, annealing for 30s at 60 ºC.

*Primers for Real-time reverse transcription (RT)-qPCR*

KLF4, forward: GCTCATGCCACCCGGTTC

reverse: CTGTGTGGGTTCGCAGGTG

HK II, forward: GGAACCCAGCTGTTTGACCA

reverse: CAGGGGAACGAGAAGGTGAAA

GLUT3, forward: CTTCCTGCTATCCTACAAAGTGC

reverse: ACTCTCATCTTTCATCTCCTGG

PGK1, forward: ATGGATGAGGTGGTGAAAGC

reverse: CAGTGCTCACATGGCTGACT

VEGF1, forward: GTCTATCAGCGCAGCTACTGC

reverse: GCCTTGGTGAGGTTTGATCCG

β2-microglobulin, forward: CCAGCAGAGAATGGAAAGTC

reverse: GATGCTGCTTACATGTCTCG

The real-time PCR was performed using ABI Prism 7000 Sequence Detection System (Applied Biosystems, Foster City, CA, USA)

*Primers for Real-time reverse transcription (RT)-qPCR for experiments involving hypoxic conditions*

HIF1α, forward: CCATTAGAAAGCAGTTCCGC

reverse: TGGGTAGGAGATGGAGATGC

HIF2α, forward: CGAACACACAAGCTCCTCTC

reverse: GTCACCACGGCAATGAAAC

VEGFA, forward: AGCCTTGCCTTGCTGCTCTA

reverse: GTGCTGGCCTTGGTGAGG

GLUT1, forward: GATTGGCTCCTTCTCTGTGG

reverse: CAGGATCAGCATCTCAAAGG

CAIX, forward: AAGAAGAGGGCTCCCTGAAG

reverse: TAGCGCCAATGACTCTGGTC

KLF4, forward: TACACAAAGAGTTCCCATCTCAAG

reverse: GTAGTGCCTGGTCAGTTCATC

HPRT, forward: TATGGCGACCCGCAGCCC

reverse: GCAAGACGTTCAGTCCTGTCCAT

*Tissue microarray (TMA)*

Antibodies:

p70S6K (Life Span Biosciences, 1:100)

Pho-P70S6K (Cell Signaling, 1:1000)

Hexokinase 2 (Cell Signaling, 1:200)

CD31 (Dako, 1:50).

*Immunoblotting*

Primary Antibodies*:*

anti-KLF4 (1:400, R&D Systems), anti-HK II (1:1000, Cell Signaling), anti-GLUT3 (1:500, LSBio), anti-PGK1 (1:1000, LSBio), anti-VEGFA (1:2000, LSBio) and anti-β-actin (1:5000, Sigma-Aldrich).

Secondary Antibodies:

HRP-Conjugated (1:5000, Cell Signaling)

Buffers:

Cell Lysis buffer: 10 mM Tris-HCl, 150 mM NaCl, 50 mM NaF, 1 mM EDTA, 1% Triton X-100, 0.1% SDS, 0.5% deoxycholate, supplemented with sodium vanadate, dithiothreitol (DTT) and protease inhibitor cocktail.

Hypoxia experiments:

Primary Antibodies:

HIF-1α (Becton Dickinson, 610958; Cayman Chemical, 10006421), HIF-2α (Novus Biologicals, NB-100-122), p70 S6 Kinase (Cell Signaling, 9202), phospho-p70 S6 Kinase (Thr389) (Cell Signaling, 9205) and Tubulin (Dianova, DLN09992) as loading controls.

Secondary Antibodies:

HRP-Conjugated (1:5,000, Dianova)

Buffer:

Cell Lysis buffer: 10 mM Tris.HCl (pH 7.5), 2% SDS, 2 mM EGTA, 20 mM NaF.

*Cell viability assay*

500 cells in 100 µl cell culture medium were seeded into 96-well plate (Sarstedt, Nümbrecht, Germany). To obtain a background luminescence signal 100 µl cell culture medium without cells were used as reference. Plate and the CellTiter-Glo® reagent were equilibrated at room temperature, and 100 µl CellTiter-Glo® reagent was added to each well and the plate was shaken for 2 min. Luminescence was measured using a TD-20/20 Luminometer (Turner Designs, Sunnyvale, CA) at indicated time points.


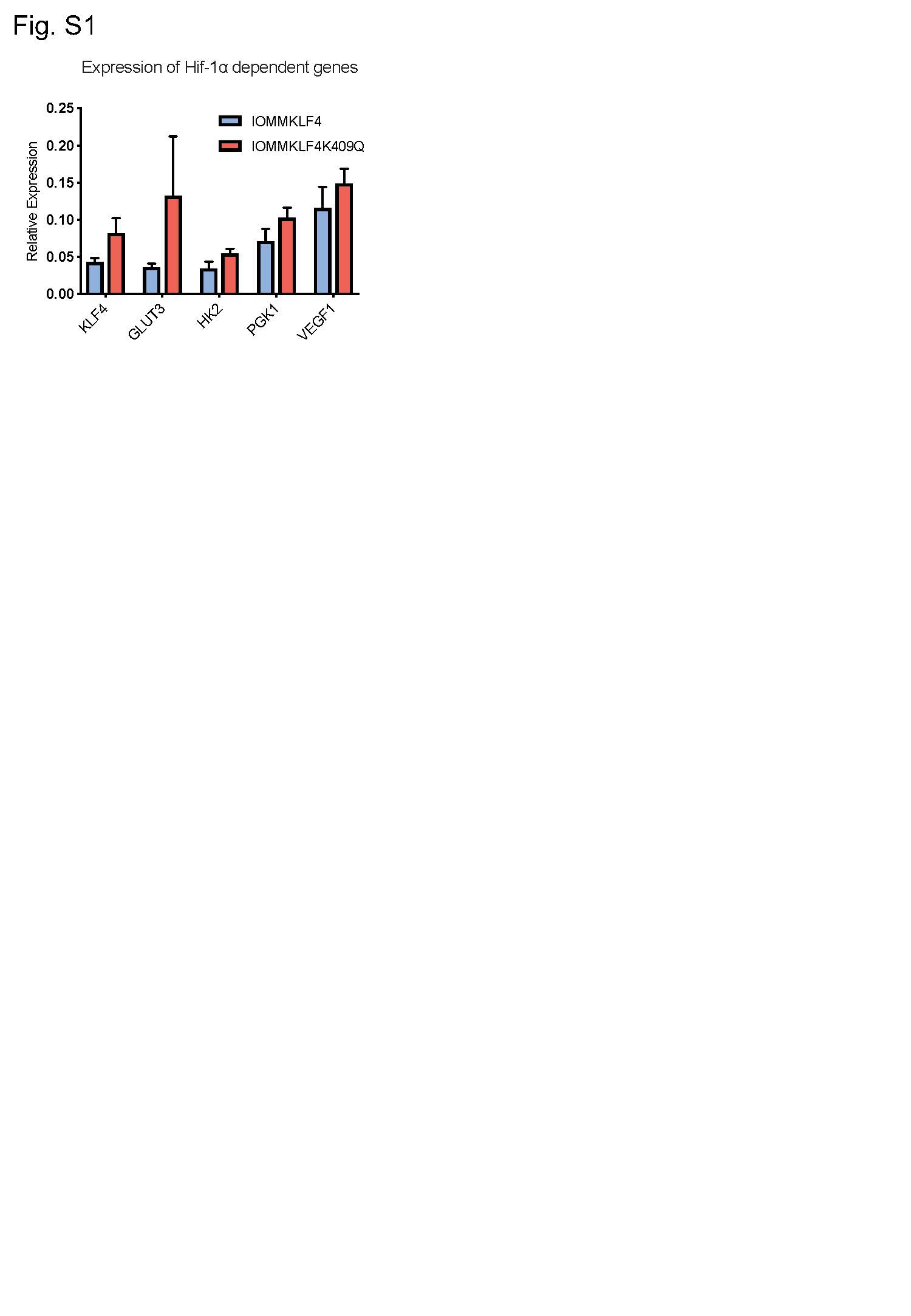


**Fig. S1** RT-qPCR analysis of HIF-1α-dependent genes in KLF4^wt/K409Q^ transfected cells (n=4)

**
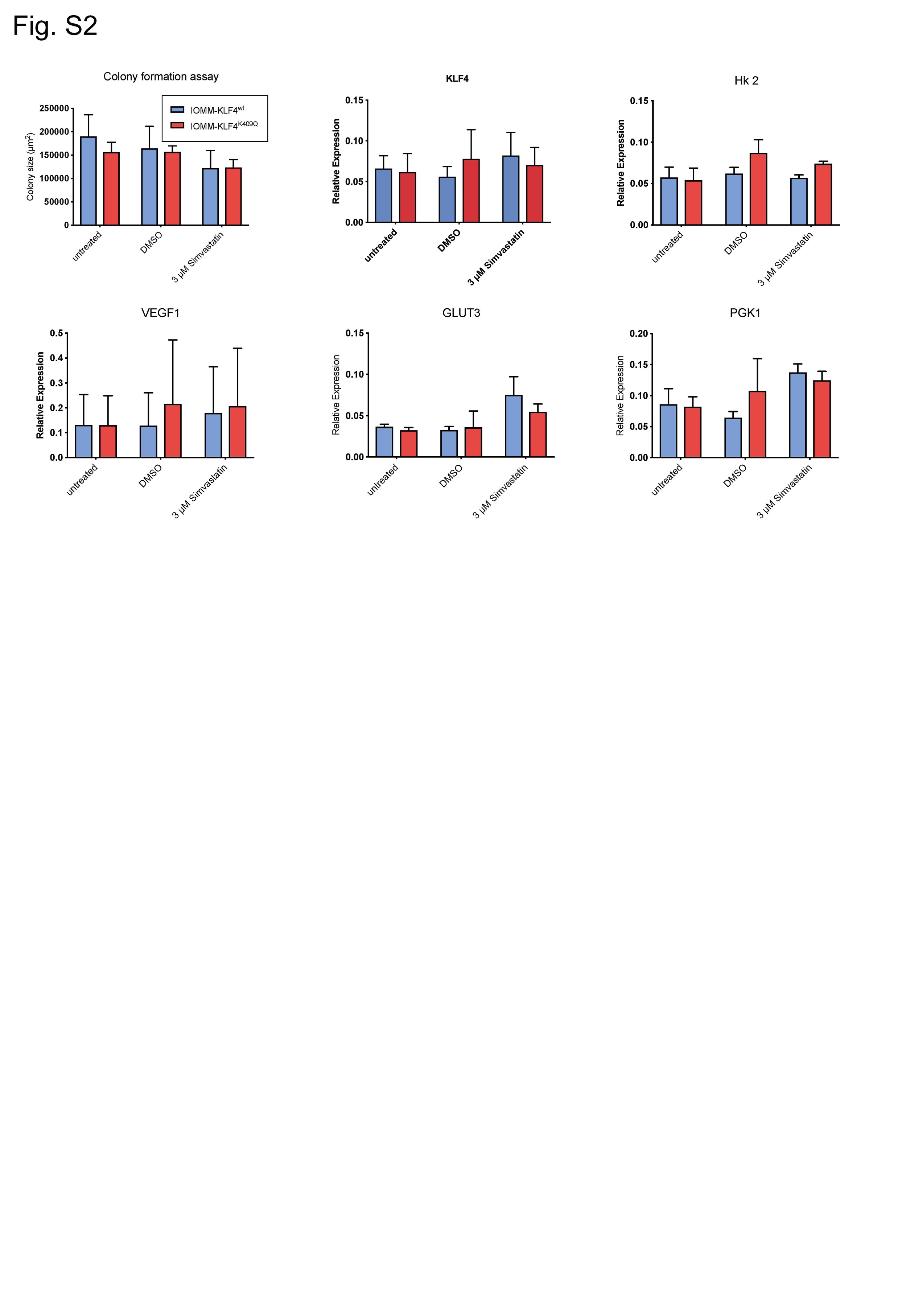
Fig. S2** Evaluation of Simvastatin treatment in IOMM-KLF4 cell lines

At a concentration of 3µM Simvastatin did not alter colony formation or KLF4/hypoxia dependent gene expression (measured through RT-qPCR) in IOMM-KLF4^wt/K409Q^ cells (n=3)

**
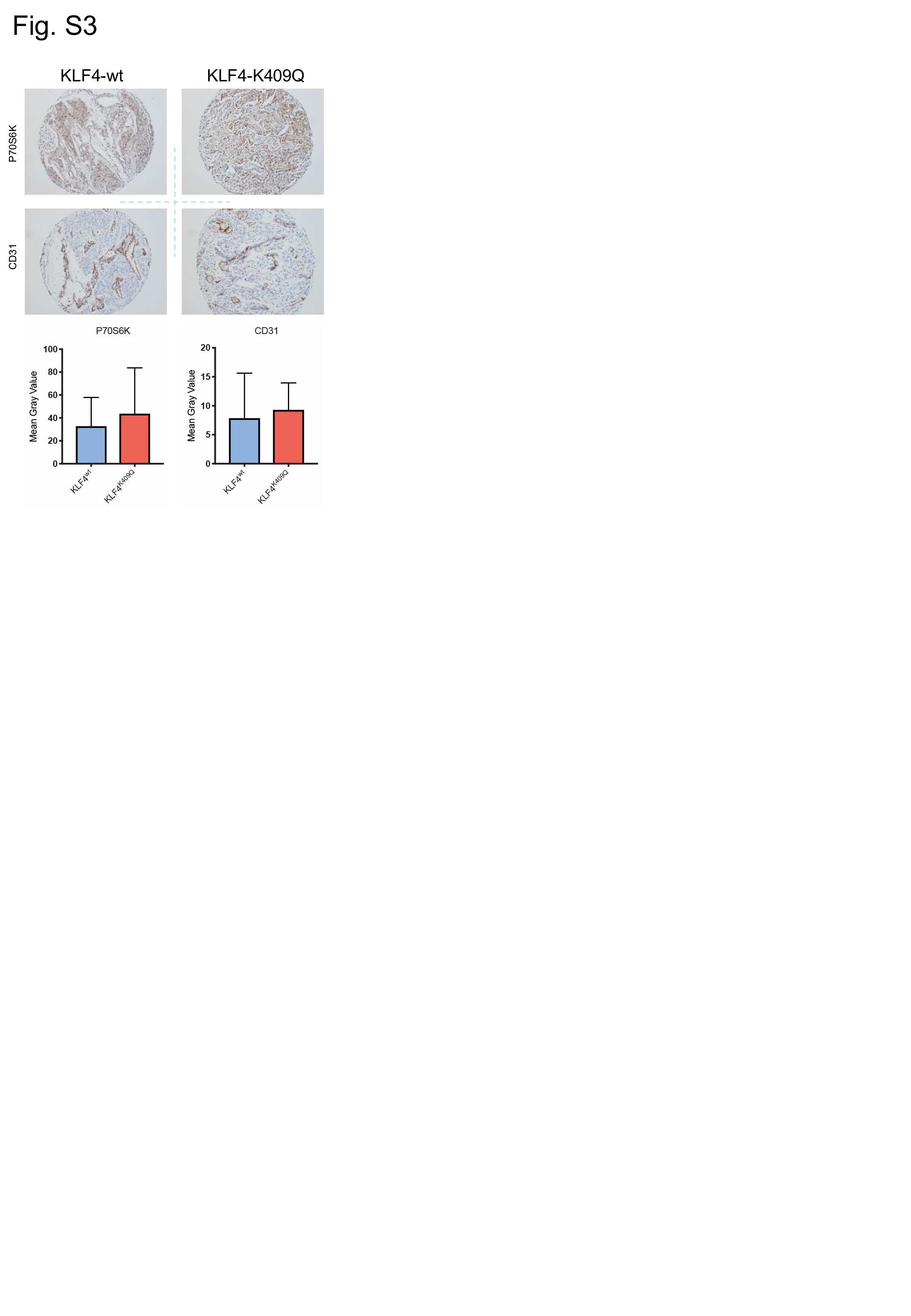
**

**Fig. S3** TMA-staining for p70S6K and CD31

(n=23*)*
